# Histopathologically confirmed radiation-induced damage of the brain – an in-depth analysis of radiation parameters and spatio-temporal occurrence

**DOI:** 10.1186/s13014-023-02385-3

**Published:** 2023-12-12

**Authors:** Mario R. P. Kossmann, Felix Ehret, Siyer Roohani, Sebastian F. Winter, Pirus Ghadjar, Güliz Acker, Carolin Senger, Simone Schmid, Daniel Zips, David Kaul

**Affiliations:** 1 Charité – Universitätsmedizin Berlin, corporate member of Freie Universität Berlin and Humboldt-Universität zu Berlin, Department of Radiation Oncology, Augustenburger Platz 1, 13353 Berlin, Germany; 2 Department of Radiotherapy and Radiation Oncology, Pius-Hospital Oldenburg, Georgstr. 12, 26121 Oldenburg, Germany; 3 Charité – Universitätsmedizin Berlin, Berlin, Germany; German Cancer Consortium (DKTK), partner site Berlin, and German Cancer Research Center (DKFZ), Heidelberg, Germany; 4 Berlin Institute of Health at Charité – Universitätsmedizin Berlin, Charitéplatz 1, 10117 Berlin, Germany; 5 Division of Neuro-Oncology, Department of Neurology, Massachusetts General Hospital, Harvard Medical School, Boston, USA; 6 Charité – Universitätsmedizin Berlin, corporate member of Freie Universität Berlin and Humboldt-Universität zu Berlin, Department of Neurosurgery, Charitéplatz 1, 10117 Berlin, Germany; 7 Charité-Universitätsmedizin Berlin, corporate member of Freie Universität Berlin and Humboldt-Universität zu Berlin, Department of Neuropathology, Charitéplatz 1, 10117 Berlin, Germany

**Keywords:** Radiation necrosis, Radionecrosis, Pseudoprogression, Subventricular zone, Heatmap, Frequency map, Location, Dose, Brain, Tumor

## Abstract

**Background:**

Radiation-induced damage (RID) after radiotherapy (RT) of primary brain tumors and metastases can be challenging to clinico-radiographically distinguish from tumor progression. RID includes pseudoprogression and radiation necrosis; the latter being irreversible and often associated with severe symptoms. While histopathology constitutes the diagnostic gold standard, biopsy-controlled clinical studies investigating RID remain limited. Whether certain brain areas are potentially more vulnerable to RID remains an area of active investigation. Here, we analyze histopathologically confirmed cases of RID in relation to the temporal and spatial dose distribution.

**Methods:**

Histopathologically confirmed cases of RID after photon-based RT for primary or secondary central nervous system malignancies were included. Demographic, clinical, and dosimetric data were collected from patient records and treatment planning systems. We calculated the equivalent dose in 2 Gy fractions (EQD2_2_) and the biologically effective dose (BED_2_) for normal brain tissue (α/β ratio of 2 Gy) and analyzed the spatial and temporal distribution using frequency maps.

**Results:**

Thirty-three patients were identified. High-grade glioma patients (n = 18) mostly received one normofractionated RT series (median cumulative EQD2_2_ 60 Gy) to a large planning target volume (PTV) (median 203.9 ccm) before diagnosis of RID. Despite the low EQD2_2_ and BED_2_, three patients with an accelerated hyperfractionated RT developed RID. In contrast, brain metastases patients (n = 15; 16 RID lesions) were often treated with two or more RT courses and with radiosurgery or fractionated stereotactic RT, resulting in a higher cumulative EQD2_2_ (median 162.4 Gy), to a small PTV (median 6.7 ccm). All (n = 34) RID lesions occurred within the PTV of at least one of the preceding RT courses. RID in the high-grade glioma group showed a frontotemporal distribution pattern, whereas, in metastatic patients, RID was observed throughout the brain with highest density in the parietal lobe. The cumulative EQD2_2_ was significantly lower in RID lesions that involved the subventricular zone (SVZ) than in lesions without SVZ involvement (median 60 Gy vs. 141 Gy, *p* = 0.01).

**Conclusions:**

Accelerated hyperfractionated RT can lead to RID despite computationally low EQD2_2_ and BED_2_ in high-grade glioma patients. The anatomical location of RID corresponded to the general tumor distribution of gliomas and metastases. The SVZ might be a particularly vulnerable area.

**Supplementary Information:**

The online version contains supplementary material available at 10.1186/s13014-023-02385-3.

## Background

Radiation-induced damage (RID) after radiotherapy (RT) of primary brain tumors and metastases can mimic tumor progression and remains a neuro-oncological dilemma [1, 2]. RID is mostly classified into pseudoprogression and radiation necrosis. Pseudoprogression occurs shortly after RT, often within weeks or months, and frequently resolves or remains stable without further therapy [1–3]. In contrast, radiation necrosis has been defined as irreversible tissue damage that occurs months or years after RT and can be associated with significant neurological morbidity and even mortality [4, 5]. However, the definitions of the terms *pseudoprogression* and *radiation necrosis* are not always consistent, leading to occasional overlap in their use.

Known risk factors for radiation necrosis include higher total radiation dose, larger fraction size and the use of concurrent and/or adjuvant systemic antineoplastic therapy [6–8]. A brain tissue tolerance dose for a 5% complication probability within 5 years from treatment (TD 5/5) for radiation necrosis of 72 Gy for normofractionated RT has been published [9]. After reirradiation, it has been reported to predominantly occur at a cumulative normalized total dose (NTD, i.e., equivalent dose in 2 Gy fractions for an α/β ratio of 2 Gy, EQD2_2_) > 100 Gy [10, 11].

The frequent need for invasive tissue biopsy to secure the diagnosis of RID has prompted substantial research efforts directed at the identification of imaging biomarkers capable of reliably distinguishing between RID and tumor progression. As such, a variety of diagnostic procedures have been explored, including standard magnetic resonance imaging (MRI) based methods, diffusion weighted imaging (DWI), perfusion-weighted imaging (PWI), magnetic resonance spectroscopy (MRS), positron emission tomography (PET), single photon emission computed tomography (SPECT), radiomics, and combinations thereof, with varying success [12–18]. Histopathology still remains the gold standard in diagnosing RID [5, 19].

Reported histopathological characteristics of radiation necrosis include coagulative and fibrinoid necrosis, gliosis, wall thickening and hyalinization of vessels, telangiectasia and calcium deposition, located predominantly in the white matter [20–22]. For pseudoprogression, similar pathologic patterns have been proposed [23], although the distinctive radiographic spatio-temporal pattern and evolution (including reversibility) suggest pathomechanistic differences to radiation necrosis [24]. Diagnosis of RID is complicated by the fact that residual vital tumor cells are often found within areas of RID [25]; moreover, *radiation-induced cellular atypia* can result in further diagnostic challenges [8, 26]. As a consequence, selective sampling may limit comprehensive histopathological assessment in the absence of robust histopathological classification criteria for RID [27].

Treatment options for radiation necrosis remain limited and include corticosteroids, bevacizumab, surgical resection, laser interstitial thermal therapy (LITT) and hyperbaric oxygen therapy (HBOT) [28, 29].

Several reports in the literature have suggested that certain areas of the brain, such as the subventricular zone (SVZ), a neural stem cell niche located along the lateral wall of the lateral ventricles [30, 31], might be more vulnerable to irradiation and thus at greater risk of developing RID [32–35]. However, studies that systematically investigate the location of RID in histopathologically confirmed cases are lacking.

Herein, we provide an in-depth analysis of 34 histopathologically confirmed RID lesions after brain-directed RT with a focus on radiation parameters and spatio-temporal distribution with specific consideration of the role of the SVZ. Furthermore, we explore putative differences between RID in patients with primary and secondary brain tumors, i.e., high-grade gliomas (HGGs) and brain metastases (BMs). The aim of this study was to correlate the radiation dose with the spatial and temporal occurrence of RID.

## Methods

### Eligibility criteria

Patients with histopathologically confirmed RID who had undergone photon RT between January 2006 and January 2020 for malignant primary and secondary brain tumors were included in this retrospective monocentric study. Patients who had received proton or heavy ion RT and those treated for benign lesions were excluded. Diagnostic tissue samples had to contain RID (with or without single vital tumor cells). All samples of “mixed lesions” containing areas of solid tumor were excluded.

The study was approved by the local institutional review board (EA2/056/20).

### Variables

Demographical, clinical, and dosimetric data, as well as imaging data were collected from the patient records and the radiation treatment planning system (TPS). The time-to-RID, defined as the interval between the day of the last fraction of RT and the first occurrence of RID on MRI imaging (new occurrence or progression of contrast-enhancement in T1-weighted gadolinium-enhanced imaging sequences, later confirmed histopathologically as RID), was noted.

### BED and EQD2 calculation

The EQD2 and biologically effective dose (BED) were calculated for RT courses in the area of RID using previously described formulas [36–39]. An α/β ratio of 2 Gy was assumed for normal brain tissue [10, 40]; this is indicated by a subscripted “2” (EQD2_2_, BED_2_).

### Delineation

Preoperative (before histopathological confirmation of RID) gadolinium-enhanced T1-weighted 3D Magnetization Prepared Rapid Acquisition with Gradient Echoes (MPRAGE) images of RID were imported into the TPS Varian Eclipse (Varian Medical Systems, Inc., Palo Alto, CA, USA). The extent of RID (defined as the contrast-enhancing lesion) was manually delineated for each patient. All contours were reviewed by a board-certified radiation oncologist, specialized in neuro-oncology (DK). The radiation plans of prior RT courses were co-registered to the MRI using rigid image registration. The planning target volumes (PTVs) were then transferred to the MRI of the RID. RT plans that clearly had no relevant dose deposition in the area of RID (e.g., in patients that were treated with radiosurgery for multiple metastases in different areas of the brain far apart from each other) were excluded. Radiation plans created in Brainlab iPlan RT (Brainlab AG, München, Germany) and Accuray Precision TPS (Accuray Incorporated, Sunnyvale, CA, USA) were exported into Varian Eclipse and then processed as described. The MRIs with the corresponding structure sets were exported in DICOM/DICOM RT format.

### Frequency map creation

The exported MRIs and structure sets were imported into 3D Slicer, version 5.0.3 [41, 42] for further processing using the Slicer RT extension [43]. Skull-stripping was performed using SynthStrip [44], included in the Freesurfer image analysis suite, version 7.3.2 [45, 46]. After minor corrections to the skull-stripping using 3D Slicer’s segment editor, bias field correction was performed using the N4ITK MRI Bias correction module [47, 48]. Lesion masks for the registration were created in segment editor for the imported RID segmentations and further lesions within the MRI (e.g., resection cavities, other metastases). The prepared MRIs were then registered to the Montreal Neurological Institute 152 (MNI152) space using the International Consortium for Brain Mapping (ICBM) 2009a nonlinear asymmetric template [49, 50] utilizing the SlicerANTs extension that is based on the Advanced Normalization Tools (ANTs) software package [51]. After an “images center of mass” initial moving transform, rigid, affine, and symmetric normalization (SyN) [52] registration were performed. The lesion masks were used for cost-function masking during the SyN registration step in most cases. For several lesions, cost-function masking resulted in worse results than performing the registration without masking. Therefore, each registration was performed with and without lesion masks and the more accurate registration was chosen after visual inspection.

The transform created during the ANTs registration was then used to register the structures (RID lesions and PTVs) to the MNI152 space. The structures were converted into binary label maps, exported and then summed using the ImCalc module of Statistical Parametric Mapping 12 (SPM12), revision 7771 [53, 54] in MATLAB (release 2022a, The MathWorks, Inc., Natick, MA, United States). The cumulative image files were imported into 3D Slicer, where they were transformed into a heatmap. This heatmap was subsequently overlaid onto the ICBM 2009a nonlinear asymmetric brain template [49, 50] for visualization.

### Lesion site mapping

In order to assign the RID lesions to the different lobes, the lobe atlas provided with the ICBM 2009a nonlinear symmetric template [49, 50] was registered to the nonlinear asymmetric template using the SlicerAnts extension of 3D Slicer. The proportion of the lesion located in the respective lobes was then calculated using the intersect function. The lesion lobe involvement was classified whenever ≥5% of the lesion was located inside the respective lobe. If a lesion extended to the area of more than one lobe, each of the involved lobes was counted for the analysis. White matter, cortex, and basal ganglia involvement was assessed by visual inspection. SVZ involvement was determined by contouring a 5 mm margin along the lateral walls of the lateral ventricles as previously described [55, 56].

### Coverage of RID lesion by PTV

The coverage of the RID by the PTVs of the single RT courses was calculated within 3D Slicer using the intersect function for each PTV. Furthermore, in order to assess the cases that had more than one preceding RT in the area of the RID, a combined PTV was computed within 3D Slicer and coverage of the RID was calculated accordingly.

### Statistical analysis

All statistical analyses were performed using IBM SPSS Statistics, version 28.0 (IBM Corp., Armonk, NY, USA). Patient characteristics for the two main subgroups, HGG and BM, were summarized using descriptive statistics. For the assessment of differences in the cumulative EQD2_2_ values between lesions with and without SVZ involvement, the Mann-Whitney U test was used. For correlation analysis, the Spearman correlation coefficient r was calculated. P values ≤ 0.05 were considered statistically significant.

## Results

A total of 33 patients with a diagnosis of RID met the inclusion criteria of our study. The diagnosis that led to brain-directed photon RT was HGG for 18 (55.5%) patients. This included glioblastoma WHO grade 4 (11 patients), oligodendroglioma WHO grade 3 (4 patients) and astrocytoma WHO grade 3 (3 patients). BMs were present in 15 (45.5%) patients. One patient in the BM group developed two independent RID lesions in different lobes of the brain. This resulted in 34 RID lesions in total. The median age at resection of the RID was 56 years (interquartile range (IQR) 49–64.5 years). Details on patient and lesion characteristics are summarized in Table 1.

**Table 1:** Patient and RID lesion characteristics

	All lesions	High-grade glioma	Brain metastasis
Number of patients	33	18 (55.5%) • Glioblastoma WHO grade 4: 11 (61.1%) • Oligodendroglioma WHO grade 3: 4 (22.2%) • Astrocytoma WHO grade 3: 3 (16.7%)	15 (45.5%) • Non-small cell lung cancer: 5 (33.3%) • Malignant melanoma: 3 (20.0%) • Renal cell carcinoma: 2 (13.3%) • Breast cancer: 2 (13.3%) • Colorectal cancer: 1 (6.7%) • Thyroid carcinoma: 1 (6.7%) • Adenocarcinoma of the esophagogastric junction: 1 (6.7%)
Median age in years at resection of first RID lesion (IQR)	56.0 (49.0–64.5)	55.5 (47.8–65.0)	56.0 (50.0–64.0)
Gender			
Female	16 (48.5%)	10 (55.6%)	6 (40.0%)
Male	17 (51.5%)	8 (44.4%)	9 (60.0%)
Median time-to-RID (interval between last RT and first occurrence of RID in MRI) in months (range)	5.8 (0.7–29.0)	5.6 (0.7–29.0)	5.8 (2.3–23.7)
Number of RID lesions	34	18 (52.9%)	16 (47.1%)
RID pathology			
RID	24 (70.6%)	10 (55.6%)	14 (87.5%)
RID + single tumor cells	10 (29.4%)	8 (44.4%)	2 (12.5%)
Type of surgery			
Resection	30 (88.2%)	15 (83.3%)	15 (93.8%)
Biopsy	3 (8.8%)	3 (16.7%)	0 (0.0%)
Autopsy	1 (2.9%)	0 (0.0%)	1 (6.3%)
Reasons for histopathological confirmation^a^			
Suspected tumor recurrence or progression or ambiguous results^b^ of the obtained imaging + symptoms associated with the lesion	21 (63.6%)	10 (58.8%)	11 (68.8%)
Suspected tumor recurrence or progression or ambiguous results^b^ of the obtained imaging without any symptoms associated with the lesion	10 (30.3%)	7 (41.2%)	3 (18.8%)
Suspected RID based on the obtained imaging, resection because of symptoms	1 (3.0%)	0 (0.0%)	1 (6.3%)
Autopsy / death of patient	1 (3.0%)	0 (0.0%)	1 (6.3%)
Data not available	1	1	0

IQR, interquartile range; MRI, magnetic resonance imaging; RID, radiation-induced damage; RT, radiotherapy

^a^ For one patient/lesion data regarding the reason for performing biopsy was not available. Percentages were calculated based on the remaining lesions (n = 33)

^b^ Tumor recurrence or progression and RID could not be definitely distinguished with the obtained imaging

### Pathology

Pathological diagnosis was made based on surgical resection (88.2%), biopsy (8.8%) or autopsy (2.9%). In 70.6% of the lesions only RID was observed, whereas in 29.4% single tumor cells were present in addition to RID. The proportion of lesions with tumor cells present was higher in the HGG subgroup (44.4% vs. 12.5%).

### Reasons for histopathological confirmation and symptoms associated with the RID lesions

The main reasons for performing resection or biopsy were suspected tumor recurrence or progression or ambiguous results of the obtained imaging, either in combination with symptoms associated with the RID lesions (63.6% of the lesions) or without any symptoms caused by RID (30.3%, Table 1). In one case, RID was suspected based on the performed diagnostic imaging (MRI and ^18^F-FET PET/CT), but the lesion was resected because of progressive symptoms. One RID lesion was diagnosed by autopsy after death of the patient from systemic tumor progression.

Additional file 1: Table S1 provides an overview of the different symptoms associated with the RID lesions in our study. The most frequent symptoms were hemiparesis (9 lesions), epileptic seizure (8 lesions) and unsteady gait (5 lesions).

### Location of RID

**Fig. 1:**
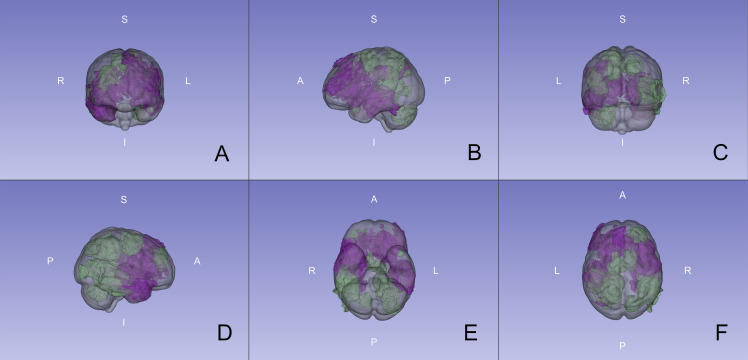
3D view of radiation-induced damage location within the brain. Model based on the International Consortium for Brain Mapping 2009a nonlinear asymmetric template. Purple: High-grade glioma subgroup. Green: Brain metastasis subgroup. View from **A** anterior, **B** left, **C** posterior, **D** right, **E** inferior, **F** superior

**Fig. 2:**
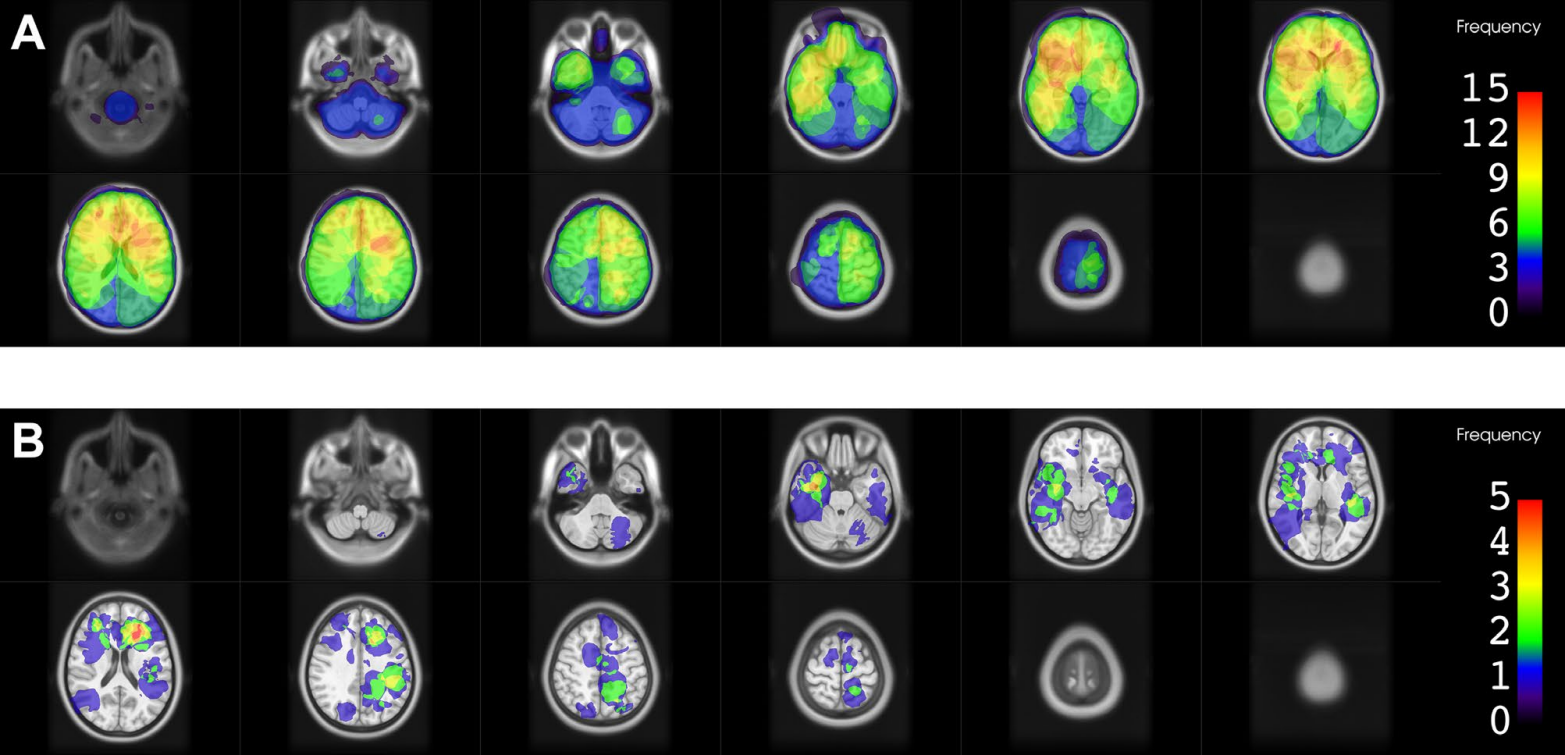
Heatmaps for planning target volumes (PTVs) and radiation-induced damage (RID) for all 33 patients combined The color bar shows the frequency of occurrence as number of contours in that location. **A**. PTVs of all radiotherapy courses in the area of RID superimposed. **B**. Frequency of occurrence of RID (contrast-enhancing lesion)

**Fig. 3:**
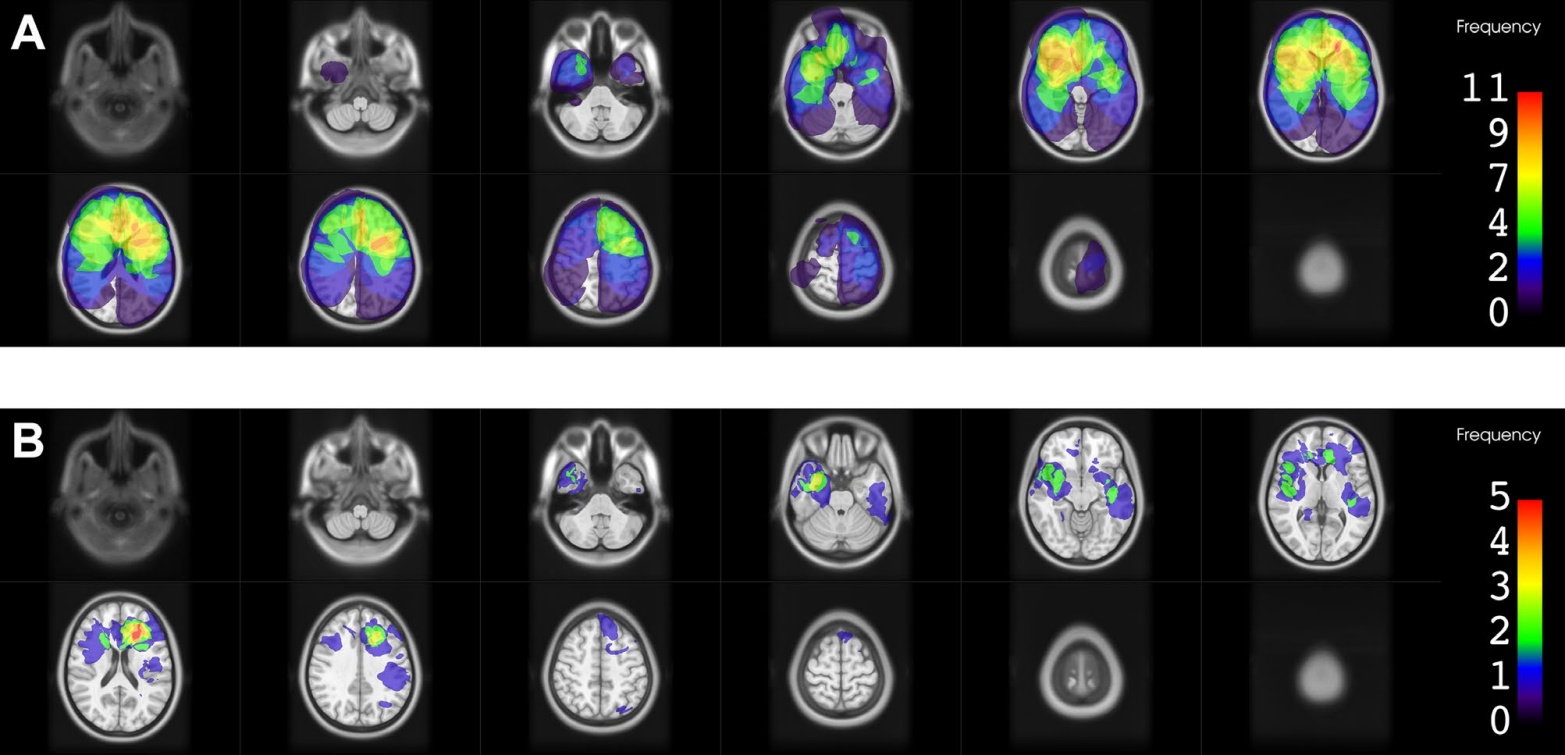
Heatmaps for planning target volumes (PTVs) and radiation-induced damage (RID) for the high-grade glioma subgroup The color bar shows the frequency of occurrence as number of contours in that location. **A**. PTVs of all radiotherapy courses in the area of RID superimposed. **B**. Frequency of occurrence of RID (contrast-enhancing lesion)

**Fig. 4:**
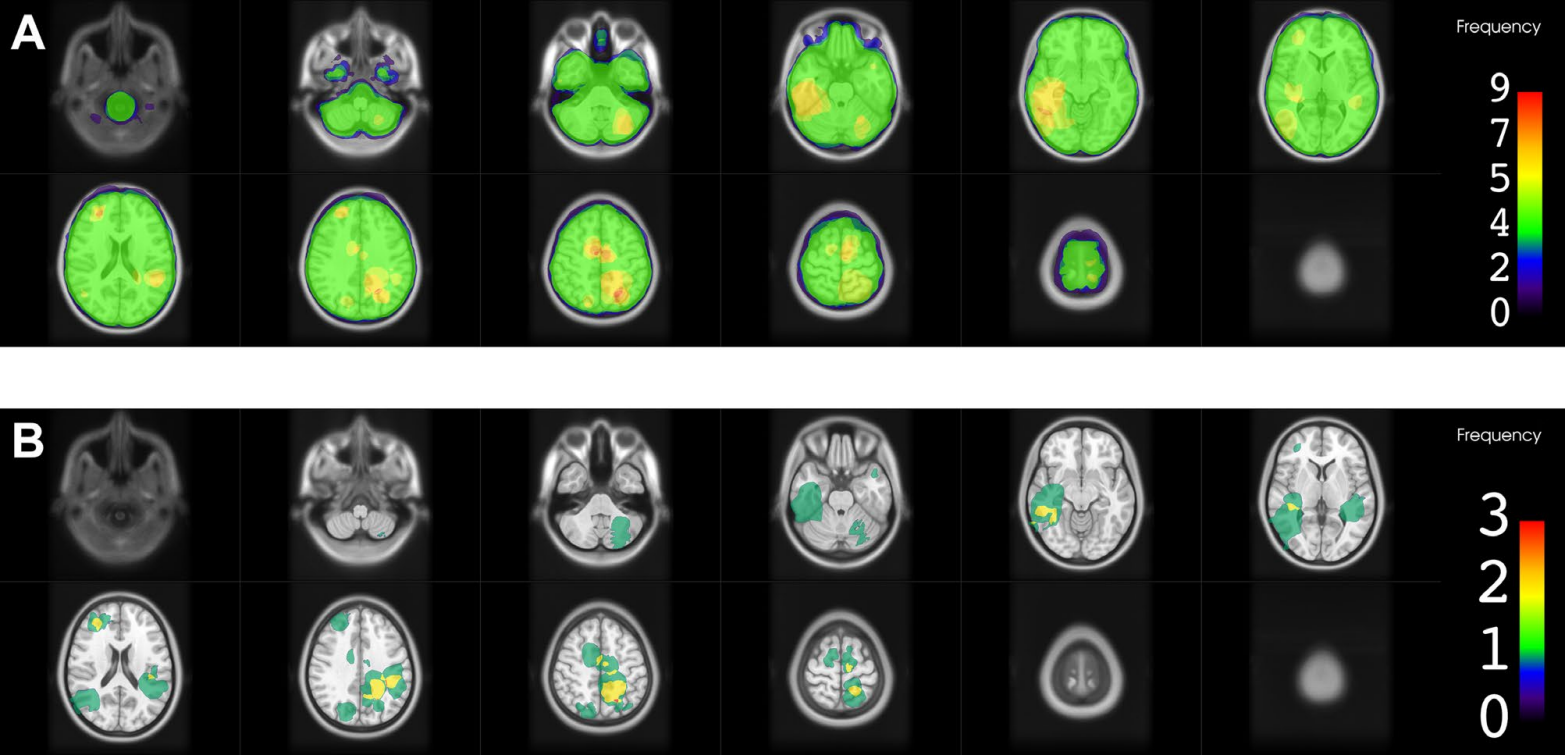
Heatmaps for planning target volumes (PTVs) and radiation-induced damage (RID) for the brain metastasis subgroup The color bar shows the frequency of occurrence as number of contours in that location. **A**. PTVs of all radiotherapy courses in the area of RID superimposed. **B**. Frequency of occurrence of RID (contrast-enhancing lesion)

A 3D model of all RID lesions separated by diagnosis is shown in Fig. 1. Figures 2, 3 and 4 show the heatmaps of the PTVs and RID lesions for the whole cohort and the respective subgroups. Classification of the lesions into the different areas of the brain is illustrated in Table 2.

All RID lesions occurred within the PTV of at least one of the preceding RT courses. The median coverage of the lesion by PTV, examined separately for each RT course, was 82.1%, with differences between the HGG subgroup (median 97.8%) and the BM subgroup (median 46.4%). The median coverage of the lesion by combined PTV, i.e., the percentage of the RID that was covered by any of the PTVs of the preceding RTs, was 97.8%, with slightly higher values in the HGG subgroup than in the BM subgroup (median 99.9% vs. 89.8%, Table 3).

Regarding the spatial distribution of RID lesions across different lobes, the frontal lobe was most commonly affected by RID among the whole cohort (55.9%) and among the HGG subgroup (72.2%). In the BM subgroup frontal lobe involvement was less frequent (37.5%), while still being the second most common location after the parietal lobe (43.8%). Overall, lesions in the BM group were more evenly distributed throughout the whole brain, whereas in the HGG subgroup no involvement of the occipital lobe and cerebellum was found. Only one lesion was found in the cerebellum in the BM group and none of the lesions were located in the brainstem.

52.9% of the lesions involved the SVZ; the proportion was higher for the HGG subgroup (77.8%) than for the BM subgroup (25.0%). All RID lesions involved the white matter, and the vast majority (94.1%) involved the cortex as well. Only two RID lesions were exclusively located in subcortical compartments, one each in the HGG and BM subgroup, respectively. Basal ganglia were involved in 29.4% of all lesions, more frequently in the HGG than in the BM subgroup (50.0% vs. 6.3%).

**Table 2:** RID lesion location

	All lesions	High-grade glioma	Brain metastasis
Hemisphere			
Left	19 (55.9%)	10 (55.6%)	9 (56.3%)
Right	14 (41.2%)	8 (44.4%)	6 (37.5%)
Bihemispheric	1 (2.9%)	0 (0.0%)	1 (6.3%)
Lobe Involvement*			
Frontal	19 (55.9%)	13 (72.2%)	6 (37.5%)
Temporal	12 (35.3%)	8 (44.4%)	4 (25.0%)
Parietal	9 (26.5%)	2 (11.1%)	7 (43.8%)
Occipital	2 (5.9%)	0 (0.0%)	2 (12.5%)
Cerebellar	1 (2.9%)	0 (0.0%)	1 (6.3%)
Subventricular zone involvement^†^	18 (52.9%)	14 (77.8%)	4 (25.0%)
Basal ganglia involvement	10 (29.4%)	9 (50.0%)	1 (6.3%)
White matter involvement	34 (100.0%)	18 (100.0%)	16 (100.0%)
Cortex involvement	32 (94.1%)	17 (94.4%)	15 (93.8%)

RID, radiation-induced damage

*only involvement of ≥5% was considered, in order to account for minor registration uncertainties

^†^lesion within a 5 mm margin along the lateral wall of the lateral ventricles

### Radiotherapy

**Fig. 5:**
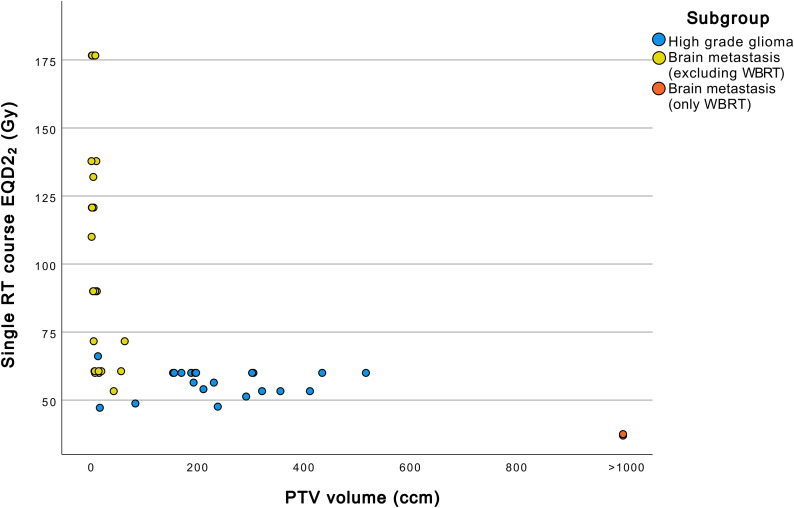
Relationship between single RT course EQD2_2_ and PTV volume ccm, cubic centimeter; EQD2_2_, equivalent dose in 2 Gy fractions (α/β ratio = 2 Gy); Gy, Gray; PTV, planning target volume; RT: radiotherapy; WBRT: whole brain radiotherapy

Table 3 summarizes RT details. In 50% of the cases a single RT course was performed in the area of the later RID. In 44.1% of the lesions two RT courses and in 5.9% three RT courses were administered, respectively. Patients in the BM subgroup received two or more RT courses more often compared to the HGG subgroup. The median cumulative EQD2_2_ administered was 103.2 Gy for the whole cohort, with higher values in the BM subgroup (median 162.4 Gy) than in the HGG subgroup (median 60 Gy). Furthermore, higher fraction size (median 8 Gy vs. 2 Gy) and single course EQD2_2_ (median 71.6 Gy vs. 60 Gy) were found in the BM subgroup. On the other hand, PTV volumes, excluding whole brain radiotherapy (WBRT), were smaller in the BM subgroup (median 6.7 ccm vs. 203.9 ccm). The relationship between single RT course EQD2_2_ and PTV volume is illustrated in Fig. 5. In 54.5% of the RTs in the HGG group a simultaneous-integrated boost (SIB) was used. In contrast, in the BM group only one boost irradiation was applied with 10 Gy in a single fraction.

The patients that received two or more RT courses all had a cumulative EQD2_2_ > 100 Gy, except of one patient with a cumulative EQD2_2_ of 98.1 Gy. The majority of RT courses (90.6%) were administered on five days per week, whereas one RT course was performed with only three fractions per week and four courses were performed with two fractions per day (b.i.d.), i.e., using an accelerated hyperfractionated RT (AHFRT) regimen, three of these in the HGG group and one in the BM group. The three AHFRT courses in the HGG group were used in patients that received only one RT course in the area of the RID and the EQD2_2_ for these lesions was among the lowest in the whole study population (53.3 Gy for each lesion).

**Fig. 6:**
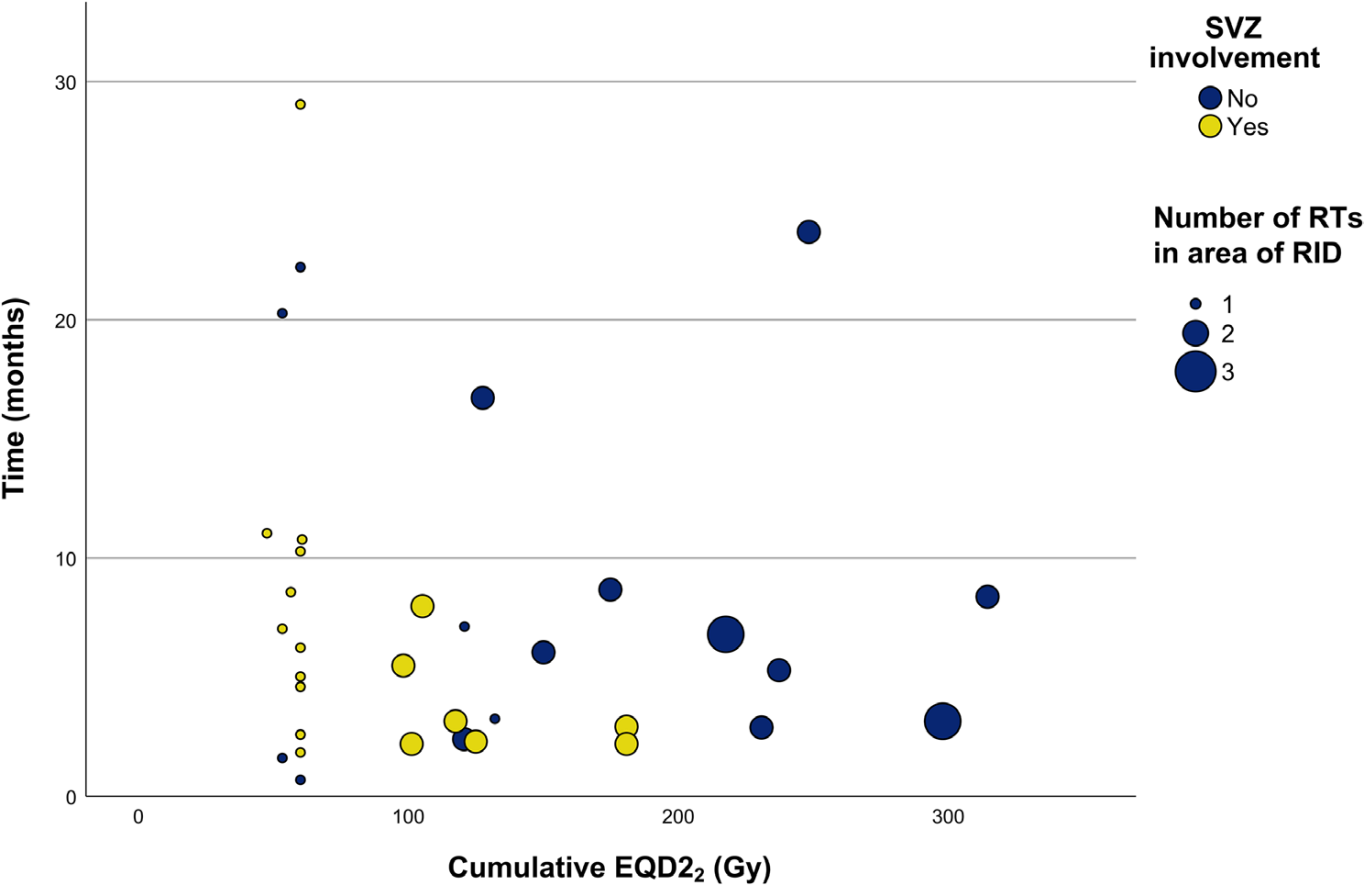
Correlation between cumulative EQD2_2_ and time-to-RID (interval between last RT and first occurrence of RID) EQD2_2_, equivalent dose in 2 Gy fractions (α/β ratio = 2 Gy); Gy, Gray; MRI, magnetic resonance imaging; RID, radiation-induced damage; RT, radiotherapy; SVZ: subventricular zone

As illustrated in Fig. 6, cumulative EQD2_2_ values were considerably lower in RID lesions that involved the SVZ (median 60 Gy, IQR 60.0–108.2 Gy) than in lesions without SVZ involvement (median 141 Gy, IQR 75.2–235.6 Gy); the differences in distributions were statistically significant (*p* = 0.01).

**Table 3:** Radiotherapy prior to RID lesion resection

	All lesions	High-grade glioma	Brain metastasis
Number of RT courses in the area of RID			
1	17 (50.0%)	14 (77.8%)	3 (18.8%)
2	15 (44.1%)	4 (22.2%)	11 (68.8%)
3	2 (5.9%)	0 (0.0%)	2 (12.5%)
Median cumulative EQD2_2_ in Gy excluding boost (IQR)	103.2 (60.0–176.3)	60.0 (55.6–70.3)	162.4 (121.8–235.6)
Median cumulative BED_2_ in Gy excluding boost (IQR)	206.4 (120.0–352.5)	120.0 (111.3–140.6)	324.8 (243.6–471.3)
Number of cases that received hypofractionation (single dose ≥2.5 Gy) in at least one RT course	20 (58.8%)	4 (22.2%)	16 (100.0%)
Median single RT course EQD2_2_ in Gy excluding boost (IQR)	60.0 (55.2–90.0)	60.0 (53.3–60.0)	71.6 (60.0–120.8)
Median number of fractions per single RT course (IQR)	11 (1–30)	30 (29.3–30.8)	3 (1–11)
Fractions per day (single RT course)			
3/week	1 (1.9%)	0 (0.0%)	1 (3.2%)
1/day	48 (90.6%)	19 (86.4%)	29 (93.5%)
2/day	4 (7.5%)	3 (13.6%)	1 (3.2%)
Median prescription dose (single RT course) in Gy (IQR)	41.8 (24.0–59.2)	59.4 (53.3–60.0)	25.6 (21.0–41.8)
Median single fraction dose in Gy (IQR)	3.8 (2.0–18.0)	2.0 (1.8–2.0)	8.0 (3.8–21.0)
Median maximum dose within PTV (single RT course) in Gy (IQR)	43.4 (30.0–63.6)	64.1 (56.1–68.3)	30.0 (26.5–43.3)
Median PTV volume in ccm excluding WBRT (IQR)*	15.9 (4.2–196.0)	203.9 (155.1–308.9)	6.7 (3.2–14.2)
Median coverage of RID lesion by single RT course PTV in percent (IQR)	82.1 (42.9–99.8)	97.8 (91.8–100.0)	46.4 (39.2–82.1)
Median coverage of RID lesion by combined PTV in percent (IQR)	97.8 (88.1–100.0)	99.9 (96.7–100.0)	89.8 (52.9–99.7)
Number of RTs with boost	13 (24.5%)	12 (54.5%)	1 (3.2%)
Median boost volume in ccm (IQR)	14.8 (7.8–72.9)	16.6 (8.8–78.2)	0.2 (*n = 1*)
Median boost EQD2_2_ in Gy (IQR)	69.3 (67.7–71.7)	69.3 (66.8–71.1)	90.6 (*n = 1*)
Prescription isodose line			
70% 80% 95%	11 (20.8%) 16 (30.2%) 26 (49.1%)	1 (4.5%) 1 (4.5%) 20 (90.9%)	10 (32.3%) 15 (48.4%) 6 (19.4%)

BED_2_, Biologically Effective Dose (α/β ratio = 2 Gy); ccm, cubic centimeter; EQD2_2_, equivalent dose in 2 Gy fractions (α/β ratio = 2 Gy); Gy, Gray; IQR, interquartile range; RID, radiation-induced damage; RT, radiotherapy; WBRT, whole brain radiotherapy

*Four cases of WBRT in the brain metastasis group were excluded from the volume analysis, as PTV volume was not available and could only be estimated for this type of RT and they were deemed to be not comparable to the other RT courses, because of very high volumes inherent to the technique of irradiating the whole brain

The median time-to-RID (interval between the end of the last RT and the first occurrence of RID on MRI imaging) was 5.8 months (range 0.7–29.0 months). There was no statistically significant correlation found between the cumulative EQD2_2_ and the time-to-RID (Fig. 6, *r*_*s*_(32) = -0.095, *p* = 0.594).

In 73.5% of the lesions systemic therapies were administered during or close to (± 1 months) the RT for at least one of the RT courses in the area of the RID. The proportion of cases receiving systemic therapy was higher in the HGG subgroup (83.5%) than in the BM subgroup (56.3%). An overview of the different drugs used is provided in Additional file 2: Table S2 in the Supplementary information.

## Discussion

The diagnosis of RID, including pseudoprogression and radiation necrosis, remains a common neuro-oncological challenge, with several important aspects to discuss. Despite its status as the diagnostic gold standard, histopathological evaluation may yield ambiguous results, especially in “mixed lesions” which include both RID and vital tumor cells and/or foci of solid tumor [6, 25, 57]. Previous works therefore vary regarding the definition of RID in presence of tumor cells and no uniform histopathological classification system has been established.

For instance, Patrizz et al. [57] classified all cases with any tumor present in addition to treatment-related changes as tumor recurrence. Campos et al. [16] required patients diagnosed with radiation necrosis to be free from recurrence for a follow-up period of ≥2 years after surgical resection. Other authors included all lesions where “treatment effect” was present, irrespective of the additional amount of tumor present [58] or chose a percentage cutoff for the ratio of recurrent tumor to radiation necrosis to classify lesions into the respective groups [59, 60].

In our study, we chose to include only cases with “pure” RID and cases with single tumor cells present, if RID accounted for most of the sample. All cases with solid tumor present in addition to RID were excluded. We deemed that this was the best compromise, as excluding all cases with tumor cells present would have led to the exclusion of almost all glioblastoma patients, but including cases with solid tumor present would have had negatively affected the diagnostic accuracy, as tumor necrosis is often present in HGG. Moreover, the potential presence of *radiation-induced cellular atypia* (reminiscent of viable tumor cells) in RID lesions constitutes another confounding factor [8, 26], circumventable by the more inclusive approach we chose.

The main reasons for performing resection or biopsy in our study were suspected tumor recurrence or progression or ambiguous results of the obtained imaging, either in combination with or without symptoms associated with the RID lesions. Two thirds of the patients developed symptoms, similar to those commonly seen in brain tumors and in some cases severe such as hemiparesis or epileptic seizures. This highlights the current diagnostic and therapeutic challenges clinicians are confronted with in the management of RID.

Regarding the temporal occurrence pattern of RID, Campos et al. [16] reported in their series of pathology-proven radiation necrosis that they found an inverse correlation between total radiation dose and the time between RT and the development of necrosis. In our study, we used the cumulative EQD2_2_ for healthy brain tissue instead, as it is better suited to reflect the differences in the fractionation schemes. Using this approach, we could not find a statistically significant correlation between the cumulative EQD2_2_ in Gy and the time-to-RID.

All RID lesions in our study occurred at least in part within the area of the PTV of one or more of the preceding RTs and in most cases coverage of RID by the PTVs was high. This is in line with the findings of Winter et al. [24] who described that in their analysis of biopsy-proven pseudoprogression and treatment-induced brain tissue necrosis in glioma patients the vast majority of the lesions occurred in the main prior radiation field.

The location pattern of RID in our study differed between the two subgroups. In the HGG subgroup RID lesions occurred preferentially in the frontotemporal areas of the brain, while posterior parts and the cerebellum were spared. If we compare our data to studies that examined the location frequency of glioma or glioblastoma, very similar location patterns are found [61–63]. Thus, the occurrence pattern of RID in this subgroup mostly seemed to follow the general location pattern of the tumors irradiated.

In the BM subgroup, RID lesions were more heterogeneously distributed throughout the whole brain in our cohort. Studies that investigated the location of BM [64, 65] also found heterogeneous distributions, while the proportion of lobe involvement differed substantially between studies and primary tumor histology. Cerebellar metastases were more common (24.6% [64] and 16.6% [65]) than the cerebellar RID in the BM subgroup in our study (one lesion, 6.3%). Whether this reflects differences in the vulnerability of the different regions of the brain or rather is caused by other factors, for instance by a less frequent usage of RT and/or surgery of the lesions in certain areas, remains unclear.

SVZ involvement of RID lesions was highly frequently observed within the HGG subgroup of our study (77.8%), substantially more often than in the BM subgroup (25.0%). However, SVZ involvement and radiation of it is more common for gliomas, too, given the necessary safety margins and respective contouring guidelines. For example, a study from our institution comprising 200 glioblastoma patients found that 66.0% of the tumors had contact to the SVZ [56]. Similarly, Roux et al. [63] found SVZ involvement in 63% of 392 patients with newly-diagnosed isocitrate dehydrogenase (IDH) wild-type supratentorial glioblastoma. Still, as discussed later, cumulative EQD2_2_ values were considerably lower in RID lesions that involved the SVZ, possibly suggesting a higher vulnerability of this region.

Patients with HGG mostly received one course of normofractionated RT or AHFRT, partially with a SIB, to a large PTV (median 203.9 ccm) with a more moderate cumulative EQD2_2_ (median 60 Gy) in comparison to the BM patients. The three patients in this subgroup that received AHFRT had relatively low EQD2_2_ (53.3 Gy) and BED_2_ (106.6 Gy) values. This was also noted in the Quantitative Analyses of Normal Tissue Effects in the Clinic (QUANTEC) publication by Lawrence et al. [9], in which the authors described a steep increase in toxicity for b.i.d. fractionation for BED > 80 Gy. One possible explanation for this might be that the shorter time interval between RT sessions allows repair of damage to the healthy brain tissue to a lesser extent. We therefore conclude that traditional dose constraints for the risk of RID might not always be universally applicable to AHFRT schemes.

BM patients, on the other hand, received two or more RT courses in more than 80% of the cases. All patients in this subgroup received hypofractionated RT (single dose ≥2.5 Gy) in at least one of their RT courses, resulting in higher cumulative (median 162.4 Gy) and single course (median 71.6 Gy) EQD2_2_ values compared to the HGG subgroup. The median PTV size was substantially smaller (6.7 ccm).

Mayer et al. [10] reported that in reirradiation trials radiation-induced normal brain tissue necrosis occurred mostly in patients receiving a cumulative NTD of > 100 Gy. A more recent review by Minniti et al. [11] covering glioblastoma reirradiation also found very low rates of radiation necrosis for patients treated with a cumulative EQD2 < 101 Gy. Our data is consistent with these results, as almost all patients in our study with the diagnosis of RID treated with more than one RT course (regardless of the subgroup) received a cumulative EQD2_2_ higher than 100 Gy, except for one patient with a cumulative EQD2_2_ of 98.11 Gy. This finding is supported by another recently published study from our institution on the reirradiation of IDH wild type glioblastoma using moderate cumulative EQD2 values that did not find any cases of confirmed radiation necrosis [66].

One possible explanation for the occurrence of RID in the HGG subgroup in spite of the lower EQD2_2_ values compared to the BM cases, might be the more frequent involvement of the SVZ in this subgroup. In the guideline for radiation necrosis published by the DEGRO in 2022 [32] it is stated that this area might be more radiosensitive due to the location of neural stem cells, a hypothesis that is supported by other studies as well [33–35]. Van West et al. [33] reported that in their cohort of low-grade glioma patients almost half of the lesions with pseudo-progressive disease were located subependymally in the ventricular wall (i.e. within the SVZ) compared to only one quarter of the lesions with real progression. In a trial of hypofractionated high-dose intensity modulated radiation therapy with concurrent and adjuvant temozolomide in patients with newly diagnosed glioblastoma, Iuchi et al. [34] pointed out that radiation necrosis was diagnosed not only at the original tumor site, but progressed more frequently and earlier in the SVZ, although this area was not in the high-dose field and doses to the SVZ were equivalent to 50 to 60 Gy of conventional RT, assuming an α/β ratio of 3. Similar findings were described by the same group in an earlier published abstract on a larger cohort of malignant astrocytomas [35]. Our work is in line with these observations, as cumulative EQD2_2_ values were significantly lower in RID lesions that involved the SVZ than in lesions without SVZ involvement. There are several possible confounders, such as the much larger PTV volumes and the frequent concurrent administration of temozolomide in HGG patients that constitute inherent risk factors for RID [6–8], but further research on the topic is warranted.

Our study has several limitations. Data collection was carried out retrospectively and at a single institution. With 33 patients and 34 lesions included, the sample size might be regarded as relatively small. However, to our knowledge, this is still one of the largest samples of RID lesions diagnosed by histopathologic confirmation. The lack of a control group without RID in this study causes some difficulties in interpreting the data, especially regarding the location analysis. Limiting the study to patients diagnosed by histopathologic confirmation bears the risk to only include the more severe and symptomatic cases of RID that had to be resected or biopsied. Furthermore, it is possible that in the location analysis RID lesions occurring in certain areas are underrepresented, because performing resection or biopsy in these areas was not clinically feasible and/or contraindicated. However, as to date there is still no other method that can provide the same diagnostic accuracy for RID as pathologic confirmation and it is still regarded the gold standard, studies based on pathology-proven samples remain an indispensable and highly valuable tool for investigating the mechanisms leading to RID after brain-directed RT.

## Conclusions

In this analysis of histopathologically confirmed RID, the location of RID mostly followed the general distribution pattern of the tumors irradiated. A previously reported correlation between the radiation dose and the period between RT and the occurrence of the RID could not be confirmed. High-grade glioma patients that receive twice-daily AHFRT schemes might develop RID despite a presumably lower EQD2_2_ and BED2_2_. The actual risk in comparison to other fractionation schemes has yet to be evaluated. Our data supports the notion that the SVZ is potentially more vulnerable to the development of RID, given that lesions there developed after a lower cumulative EQD2_2_. The larger irradiated volumes as well as the more frequently performed concurrent administration of temozolomide in these lesions might have to be considered as independent risk factors for RID; thus, further research on the topic should ideally address these potential confounders.

### Electronic supplementary material

Below is the link to the electronic supplementary material.

Supplementary Material 1

Supplementary Material 2

## Data Availability

Due to data protection regulations, the data analyzed for this publication cannot be shared on a publicly available repository.

## Abbreviations

AHFRT: Accelerated hyperfractionated radiotherapy
ANTs: Advanced Normalization Tools
BED: Biologically effective dose
b.i.d: Bis in die (twice a day)
BM: Brain metastasis
ccm: Cubic centimeter
DEGRO: German Society for Radiation Oncology (Deutsche Gesellschaft für Radioonkologie)
DWI: Diffusion weighted imaging
EQD2: Equivalent dose in 2 Gy fractions
Gy: Gray
HGG: High-grade glioma
IDH: Isocitrate dehydrogenase
MPRAGE: Magnetization Prepared Rapid Acquisition with Gradient Echoes
IQR: Interquartile range
MRI: Magnetic resonance imaging
MRS: Magnetic resonance spectroscopy
NTD: Normalized total dose
PET: Positron emission tomography
PWI: Perfusion-weighted imaging
RID: Radiation-induced damage
PTV: Planning target volume
QUANTEC: Quantitative Analyses Of Normal Tissue Effects In The Clinic
RT: Radiotherapy
SPECT: Single photon emission computed tomography
SPM12: Statistical Parametric Mapping 12
SVZ: Subventricular zone
SyN: Symmetric normalization
TD 5/5: Tolerance dose for a 5% complication probability within 5 years from treatment
TPS: Treatment planning system
WBRT: Whole brain radiotherapy
