# Supplementary material for: Histopathologically confirmed radiation-induced damage of the brain – an in-depth analysis of radiation parameters and spatio-temporal occurrence
Source: Radiat Oncol. 2023 Dec 12;18:198. doi: 10.1186/s13014-023-02385-3 (PMC10717523; doi:10.1186/s13014-023-02385-3)
Supplement: Supplementary file 1 — Supplementary Material 1 [file 13014_2023_2385_MOESM1_ESM.docx]

**Table S1:** Symptoms associated with the RID lesions

| Symptom | Number of lesions |
| --- | --- |
| Hemiparesis | 9 |
| Epileptic seizure | 8 |
| Unsteady gait | 5 |
| Dysarthria | 4 |
| Memory disorder | 4 |
| Aphasia | 3 |
| Tendency to fall | 3 |
| Vertigo | 3 |
| Disorientation | 2 |
| Dysdiadochokinesis | 2 |
| Headache | 2 |
| Hypoesthesia | 2 |
| Frontal lobe syndrome | 2 |
| Psychomotor retardation | 2 |
| Apraxia | 1 |
| Arm paresis | 1 |
| Dysmetria | 1 |
| Formal thought disorder | 1 |
| Hemispatial neglect | 1 |
| Impaired consciousness | 1 |
| Impaired coordination | 1 |
| Paresthesia | 1 |
| Phantogeusia | 1 |
| Poor concentration | 1 |
| Visual impairment | 1 |

For 23 lesions / 22 patients symptoms associated with the RID were reported.

RID, radiation-induced damage
