# Supplementary material for: Histopathologically confirmed radiation-induced damage of the brain – an in-depth analysis of radiation parameters and spatio-temporal occurrence
Source: Radiat Oncol. 2023 Dec 12;18:198. doi: 10.1186/s13014-023-02385-3 (PMC10717523; doi:10.1186/s13014-023-02385-3)
Supplement: Supplementary file 2 — Supplementary Material 2 [file 13014_2023_2385_MOESM2_ESM.docx]

**Table S2:** Use of anti-cancer drugs prior to resection of radiation-induced damage

|  | All lesions | High-grade glioma | Brain metastasis |
| --- | --- | --- | --- |
| Received anti-cancer drugs during or close to (±1 months) RT | 25 (73.5%) | 16 (88.9%) | 9 (56.3%) |
| Type of anti-cancer drug |  | Temozolomide alone: 13 (81.3%)  Temozolomide + CCNU: 1 (6.3%)  Temozolomide + Cilengitide: 1 (6.3%)  Temozolomide (RT1) // Procarbazin + CCNU (RT2): 1 (6.3%) | Paclitaxel + Ramirucumab: 1 (11.1%)  Cisplatin + Pemetrexed + Bevacizumab: 1 (11.1%)  Nivolumab: 1 (11.1%)  Capecitabine: 1 (11.1%)  Lapatinib + Herceptin: 2 (22.2%)  Carboplatin + Gemcitabine: 1 (11.1%)  Atezolizumab: 1 (11.1%)  Fulvestrant (RT1) // Letrozol + Palbociclib (RT2): 1 (11.1%) |

RT, radiotherapy; RT1, first course of radiotherapy; RT2, second course of radiotherapy
